# Pediatric Fabry disease revealed by early neuropathic symptoms: a case report

**DOI:** 10.1093/omcr/omag191

**Published:** 2026-10-05

**Authors:** Imane Mezdaoui, Abdelilah Radi, Sara Babour, Azzeddine Laaraje, Amale Hassani, Rachid Abilkassem

**Affiliations:** Department of Pediatrics, Military Hospital Mohammed V, Avenue des Forces Armées Royales, Hay Riad, Rabat 10000, Morocco; Department of Pediatrics, Military Hospital Mohammed V, Avenue des Forces Armées Royales, Hay Riad, Rabat 10000, Morocco; Department of Pediatrics, Military Hospital Mohammed V, Avenue des Forces Armées Royales, Hay Riad, Rabat 10000, Morocco; Department of Pediatrics, Military Hospital Mohammed V, Avenue des Forces Armées Royales, Hay Riad, Rabat 10000, Morocco; Department of Pediatrics, Military Hospital Mohammed V, Avenue des Forces Armées Royales, Hay Riad, Rabat 10000, Morocco; Department of Pediatrics, Military Hospital Mohammed V, Avenue des Forces Armées Royales, Hay Riad, Rabat 10000, Morocco

**Keywords:** Fabry disease, lysosomal storage disorder, enzyme replacement therapy, pediatrics, genetic mutation

## Abstract

Fabry disease (FD) is a rare X-linked lysosomal storage disorder caused by α-galactosidase A deficiency, resulting in progressive glycosphingolipid accumulation and multisystem morbidity. Early pediatric manifestations are frequently overlooked, delaying treatment and increasing the risk of irreversible organ damage. We describe a 14-year-old boy presenting with acroparesthesia, recurrent abdominal pain, exercise intolerance, and anhidrosis since early childhood. Clinical evaluation revealed peripheral neuropathy and short stature. Biochemical testing demonstrated nearly absent α-galactosidase A activity (0.1 μmol/L/h; N > 2.8) and markedly elevated lyso-GL-3 (135.6 ng/mL; N < 3.5). Molecular analysis identified a hemizygous pathogenic variant, c.658C>T (p.Arg220^*^), confirming classic FD. Comprehensive baseline assessment showed no renal, cardiac, ophthalmologic, or cerebrovascular involvement. Familial screening revealed the same mutation in his mother and a symptomatic maternal uncle. Enzyme replacement therapy (ERT) with agalsidase beta was initiated. Early recognition in childhood is critical to optimize outcomes and modify disease progression.

## Introduction

Fabry disease (FD) is a progressive X-linked lysosomal storage disorder caused by deficient α-galactosidase A (α-Gal A) activity, leading to accumulation of globotriaosylceramide (GL-3) and lyso-GL-3 in multiple tissues [1–3]. The classic phenotype presents in childhood with acroparesthesia, gastrointestinal disturbances, hypohidrosis, and heat intolerance, later progressing to renal, cardiac, and cerebrovascular complications if untreated [2, 4].

Early symptoms are often nonspecific, resulting in delayed diagnosis [5]. Measurement of α-Gal A activity, lyso-GL-3 levels, and confirmatory GLA gene sequencing establish the diagnosis [3, 6]. Enzyme replacement therapy (ERT) can slow disease progression, particularly when initiated before irreversible organ damage occurs [7].

We report a pediatric case of classic FD confirmed by a pathogenic GLA mutation, highlighting the importance of early recognition and family screening.

## Case report

A 14-year-old male was referred for chronic neuropathic pain, abdominal discomfort, and reduced exercise tolerance. Symptoms began at age four with intermittent burning pain in the hands and feet, exacerbated by heat and exertion. He later developed anhidrosis and fatigue. These complaints were initially attributed to functional causes.

He was born at term with normal neonatal history. He was the first child of third-degree consanguineous parents. Family history revealed maternal relatives with early renal disease and cardiomyopathy of unknown origin.

On examination, he had short stature (155 cm, 37 kg). Neurological evaluation showed decreased pinprick and vibration sensation in the lower limbs. No angiokeratomas or corneal verticillata were observed. Cardiovascular, abdominal, and respiratory examinations were normal.

Laboratory testing demonstrated markedly reduced plasma α-Gal A activity (0.1 μmol/L/h; normal > 2.8) and elevated lyso-GL-3 (135.6 ng/mL; normal < 3.5). Genetic analysis identified a hemizygous nonsense mutation in GLA: c.658C>T (p.Arg220^*^), confirming classic FD.

Baseline evaluation including renal function, 24-hour proteinuria, electrocardiography, echocardiography, brain MRI, and ophthalmologic and audiologic assessments showed no evidence of organ involvement.

ERT with agalsidase beta (1 mg/kg every two weeks) was initiated. Treatment was well tolerated without infusion reactions. At follow-up, the patient reported improvement in neuropathic pain and exercise tolerance. No new organ involvement was detected.

Family screening identified the same hemizygous variant in a symptomatic 31-year-old maternal uncle (undetectable α-Gal A activity, lyso-GL-3 93.6 ng/mL) and heterozygous carriage in his mother, emphasizing the importance of cascade testing.

## Discussion

FD results from GLA mutations causing deficient α-Gal A activity and progressive glycosphingolipid accumulation in endothelial, renal, cardiac, and neuronal tissues [2, 5]. The estimated incidence ranges from 1:40000 to 1:117000 live male births, though newborn screening suggests underdiagnosis [1–4].

Classic FD typically manifests in childhood with neuropathic pain, gastrointestinal symptoms, and autonomic dysfunction [1, 3]. Without treatment, progressive renal failure, hypertrophic cardiomyopathy, arrhythmias, and cerebrovascular events occur in adulthood [1, 6]. Our patient exhibited characteristic early symptoms but experienced a significant diagnostic delay.

Diagnosis relies on reduced α-Gal A activity and elevated lyso-GL-3, with genetic confirmation [2, 3]. The identified nonsense mutation (p.Arg220^*^) produces a truncated nonfunctional enzyme consistent with the classic phenotype.

Early initiation of ERT is associated with improved pain control, stabilization of renal function, and delayed cardiac complications [7, 8]. Initiating therapy before organ damage is critical, as established fibrosis is largely irreversible. In our case, treatment was started prior to detectable organ involvement, which may improve long-term prognosis.

Limitations of ERT include incomplete blood–brain barrier penetration, antibody development, and high cost [6, 7]. Emerging therapies such as pharmacological chaperones, substrate reduction therapy, and gene therapy are under investigation [2, 5].

The novelty of this observation lies not in the phenotype, which is typical, but in the diagnostic trajectory. Despite symptom onset at four years of age and a nonsense variant predicting a classic phenotype, the patient reached adolescence with no detectable renal, cardiac, cerebrovascular, ophthalmologic or audiologic involvement, leaving an unusually preserved therapeutic window after a ten-year diagnostic delay. Newborn screening allows presymptomatic detection, but it is implemented in only a small number of programmes worldwide, including Taiwan, Japan, parts of Italy and several United States programmes [9]. In Morocco, Fabry disease is not screened at birth, national newborn screening infrastructure remains limited, and consanguinity is frequent [10]. This gap plausibly contributed to the delay observed here, and cascade testing subsequently identified two additional affected relatives from a single index case, supporting consideration of Fabry disease for inclusion in expanded newborn screening programmes.

This case underscores the importance of recognizing neuropathic pain and heat intolerance in children as potential early signs of FD. Careful family history and biochemical testing can facilitate timely diagnosis and intervention.

Early recognition of Fabry disease in children can significantly alter disease progression. This case illustrates that prompt biochemical and genetic evaluation in patients with unexplained neuropathic symptoms enables early treatment initiation, potentially preventing irreversible organ damage.
